# 

**Published:** 1822-02

**Authors:** 


### MONTHLY CATALOGUE OF MEDICAL BOOKS.

**A System of Materia Medica and Pharmacy; including Translations of the London, Edinburgh, and Dublin Pharmacopœiæ. By John Murray, M.D. Fourth Edition. 2 vols. 8vo. 1l. 4s.**

**Elements of Chemistry. By John Murray, M.D. In two vols. 8vo. 1l. 5s.**

**A Dictionary of Practical Surgery; comprehending all the most interesting Improvements from the earliest times down to the present period; an Account of the Instruments, Remedies, and Applications, employed in Surgery; the Etymology and Signification of the principal Terms; and numerous References to ancient and modern Works. By Samuel Cooper, M.R.C.S. formerly Surgeon to the Forces. New Edition, greatly improved and enlarged. 8vo. 27s. boards.**

**The Principles of Medicine, on the Plan of the Baconian Philosophy. Vol. I. on Febrile and Inflammatory Diseases. By R. D. Hamilton. 8vo. 9s.**

**An Epitome of Pharmaceutical Chemistry, whereby the Art of Prescribing scientifically may be facilitated, and those Decompositions avoided which often frustrate the Views of the Practitioner in their Medical Effects. By Rees Price, M.D. 3s.**

**The Edinburgh New Dispensatory. By Andrew Duncan, jun. M.D.**

**On the ancient and modern Opinions on Life and Organization. By John Barclay, M.D.**

**A Letter to the Right Hon. the Lord Provost, as Chairman of the Court of Contributors to the Royal Infirmary of Edinburgh. By Robert Liston, Surgeon.**

---

### NOTICES TO CORRESPONDENTS.

**Dr. Kinglake's** ***second Paper is postponed till our next, for want of room.***

***We beg to return our thanks to*** **Mr. Greenhow,** ***of Newcastle, for his Commucation.***

**"A Well-Wisher"** ***to our Journal will perceive that, under the head of*** **Academical Proceedings,** ***we have adopted his suggestion.***

***We are, this month, so overwhelmed with engagements of various descriptions, that we must apologize to our Correspondents, "friends and enemies," for the abrupt manner in which we crave leave to decline noticing their*** **favours** ***at present.***

**H. S., R. H., *and* T. W.,** ***may rest assured, that we have not lost sight of the chace. The fox is in sight, and they shall be in at the death of it.***

---

### NOTICE.

*The Proprietors beg to acquaint the Medical Profession, that they have a few Copies remaining of the* GENERAL INDEX, *which comprises an Analytical Table of Contents, from* Volume One to Forty *inclusive; carefully arranged in Alphabetical Order, with References to the whole of the cited Authorities, under their nominal Characters, &c. and forming an indispensable* Appendix *to the Journal; in one large* 8vo. *volume, price* 21*s.*

